# Exploring Antioxidant and α-Glucosidase Inhibitory Activities in Mulberry Leaves (*Morus alba* L.) across Growth Stages: A Comprehensive Metabolomic Analysis with Chemometrics

**DOI:** 10.3390/molecules29010171

**Published:** 2023-12-27

**Authors:** Wenjie Li, Shenghui Hao, Hengyang Li, Qi An, Lina Yang, Bing Guo, Zijing Xue, Yongli Liu, Long Guo, Yuguang Zheng, Dan Zhang

**Affiliations:** 1 Traditional Chinese Medicine Processing Technology Innovation Centre of Hebei Province, College of Pharmacy, Hebei University of Chinese Medicine, Shijiazhuang 050200, China; 17370230043@163.com (W.L.); hshskctbdh@163.com (S.H.); lhy56778@163.com (H.L.); yanglina@hebcm.edu.cn (L.Y.); guob1128@163.com (B.G.); 18931365812@163.com (Z.X.); guolong@hebcm.edu.cn (L.G.); 2 Department of Chinese Materia Medica, Hebei Institute for Drug and Medical Device Control, Shijiazhuang 050200, China; 18233106330@163.com (Q.A.); liuyongli2008@126.com (Y.L.); 3 Department of Pharmaceutical Engineering, Hebei Chemical and Pharmaceutical College, Shijiazhuang 050026, China

**Keywords:** *Morus alba* L., primary metabolites, secondary metabolites, different growth stages, antioxidant activity

## Abstract

Metabolic product accumulation exhibited variations among mulberry (*Morus alba* L.) leaves (MLs) at distinct growth stages, and this assessment was conducted using a combination of analytical techniques including high-performance liquid chromatography (HPLC), gas chromatography–mass spectrometry (GC-MS), and liquid chromatography–mass spectrometry (LC-MS). Multivariate analysis was applied to the data, and the findings were correlated with antioxidant activity and α-glucosidase inhibitory effects in vitro. Statistical analyses divided the 27 batches of MLs at different growth stages into three distinct groups. In vitro assays for antioxidant activity and α-glucosidase inhibition revealed that IC_50_ values were highest at the Y23 stage, which corresponds to the ‘Frost Descends’ solar term. In summary, the results of this study indicate that MLs at different growth stages throughout the year can be categorized into three primary growth stages using traditional Chinese solar terms as reference points, based on the observed variations in metabolite content.

## 1. Introduction

*Morus alba* L., commonly known as mulberry, is a deciduous tree that has exhibited extensive distribution throughout China since antiquity. Because of its ability to adapt to many different climatic conditions, it is now widely grown in many countries around the world [1]. Mulberry leaves (MLs) serve a dual purpose, being utilized both as a primary food source for silkworms in sericulture and as a herbal medicine [2]. MLs were first recorded in “Shen Nong’s Herbal Classic” as “Shen Xian Ye” [3]. In Chinese medicine, it can be used to treat wind-heat colds, lung-heat and dry cough, dizziness, headache, etc. Abundant in MLs are a diverse array of bioactive and nutritional compounds, encompassing flavonoids, amino acids, α-aminobutyric acid, vitamins, polysaccharides, alkaloids, and steroids, as demonstrated by numerous studies [4,5,6]. MLs possess a wide range of pharmacological effects, especially in antioxidation, hypoglycemia, anti-cholesterol, anticancer, antiinflammation and others [7]. MLs can also be made into ML tea, ML wine, ML vinegar, ML noodles, and other foods, in addition to being a traditional Chinese medicine. In 1993, the Ministry of Health of China recognized MLs as the first batch of herbal medicines of the same source as food. Owing to the wide range of bioactivities, its application in the fields of medicine, food, and healthcare products has great development space and application prospects.

Multiple factors, including nutritional composition, functional constituents, and antioxidant potential, collectively influence the quality of MLs. These aspects collectively impact the medicinal and economic value of MLs. MLs harvested in different seasons serve diverse purposes. MLs collected during spring, summer, and autumn are primarily used for silkworm rearing and food processing. In China, there are 24 solar terms representing changes in natural rhythms, marking the establishment of the “12-month construction” in the calendar. These solar terms hold profound cultural significance deeply rooted in Chinese historical heritage. They accurately mirror shifts in natural phenomena and play a pivotal role in people’s daily lives. Traditionally, these 24 solar terms serve as crucial agricultural and harvesting milestones, often indicating the timing for planting crops, exemplified by specific solar terms as indicators for sowing seeds. In contrast, MLs harvested after the initial frost are typically reserved for medicinal applications. The content of flavonoids in MLs reaches its peak during the period following the first frost and extending into November [8]. Additionally, another study investigated the correlation between the accumulation of total flavonoids and ambient temperature, suggesting that lower temperatures promote the accumulation of flavonoid compounds [9]. Furthermore, investigations have demonstrated that MLs affected by frost exhibit a significant increase in antioxidant activity and chlorogenic acid content [10]. While various researchers have arrived at a consistent conclusion regarding the superior quality of MLs after exposure to frost, limited attention has been given to the accumulation of primary and secondary metabolites throughout the entire growth stages of MLs. The primary and secondary metabolites of MLs exhibit significant variations across different growth stages. Consequently, we conducted a study to investigate the differences in metabolites at various growth stages of MLs. 

Metabolomics has emerged as an increasingly utilized analytical approach for assessing the quality of both food and herbal products, ensuring their quality through targeted and untargeted methods [11]. While untargeted analysis aims to comprehensively analyze all measurable metabolites within a sample, encompassing both known and unknown compounds, targeted metabolomics narrows its focus to specific metabolites of interest [12]. This approach enables the visualization, investigation, and comprehension of metabolite changes within a plant’s metabolome, resulting from environmental perturbations or inherent differences in metabolic profiles due to natural or altered states [13]. Liquid chromatography–mass spectrometry (LC-MS) and gas chromatography coupled to mass spectrometry (GC-MS), valued for their high sensitivity and accuracy, have now become standard analytical platforms extensively employed in plant metabolomics research [14]. GC-MS is suitable for analyzing primary metabolites such as organic acids, sugars, and amino acids after derivatization to enhance volatility. However, most secondary metabolites are thermally labile and unsuitable for GC-MS. LC-MS, on the other hand, can overcome this limitation [15]. Within chemometric techniques, principal component analysis (PCA) serves as an unsupervised pattern-recognition method, facilitating the visualization of grouping trends and identification of outliers. Orthogonal partial least squares–discriminant analysis (OPLS-DA), on the other hand, acts as a supervised pattern-recognition method capable of analyzing, classifying, and reducing the dimensionality of complex datasets. Cluster analysis (CA), a multivariate statistical method, is employed for sample or indicator classification. Additionally, a heatmap was utilized to depict the relative concentration trends of compounds across all samples. These techniques are widely employed for exploratory data overview and further discrimination purposes, respectively [16,17].

In recent years, there has been a growing recognition of the medicinal and economic value of MLs. However, a comprehensive investigation into the metabolites of MLs and their variations at different growth stages has been lacking. In this study, we employed GC-MS and LC-MS technologies in conjunction with multivariate data analysis to examine the differences in metabolite profiles of MLs across various growth stages. Furthermore, we assessed the antioxidant and α-glucosidase inhibitory activity in vitro to evaluate how MLs’ bioactivity varies with growth stages. The outcomes of this research are anticipated to enhance our comprehension of the biochemical mechanisms underlying ML development. Simultaneously, this study serves as a foundational step towards the efficient utilization and advancement of ML resources in both medicinal and economic domains.

## 2. Results and Discussion

For the comparative assessment of datasets obtained from various metabolomic platforms, including ultra-performance liquid chromatography (UPLC)-MS and GC-MS metabolomics, a standardized extraction method was developed, as described in Section 3.2 regarding sample preparation for both MS and HPLC analyses. Chemometric methods were applied to categorize the samples, ensuring analytical uniformity, and revealing their shared characteristics as well as differences.

### 2.1. GC-MS Profiling of Metabolites in ML Extract

#### 2.1.1. GC-MS Profiling of Metabolites

**Figure 1:**
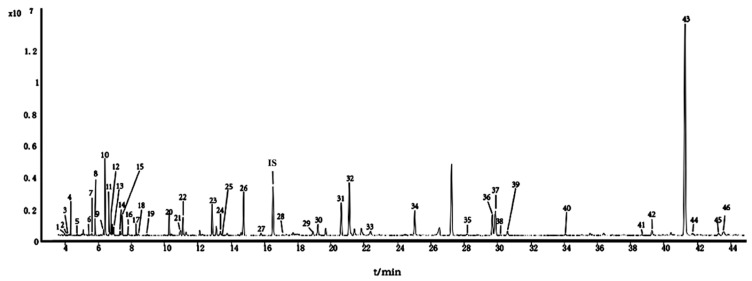
Representative GC-MS chromatograms of trimethylsilyl (TMS) derivatives of primary metabolites from mulberry leaves (MLs).

GC-MS analysis following post-silylation was employed to generate a comprehensive profile of primary metabolites from MLs at different growth stages. The reproducibility of the fragment patterns in the GC-MS experimental data was reliable [18] and aligned well with the National Institute of Standards and Technology 17 (NIST17) library. A total of 46 metabolites (Figure 1 and Table 1) were identified, including organic acids, sugars, and amino acids.

#### 2.1.2. Multivariate Data Analysis of MLs at Different Growth Periods

Xylitol was chosen as a reference peak for the validation of the method concerning metabolites in ML extracts. The relative peak area (RPA) for each compound was determined using the area normalization approach. To gain deeper insights into the relative variations in metabolite content across different growth stages of MLs, several multivariate statistical techniques were applied, including PCA, OPLS-DA, and CA, for data analysis.

**Figure 2:**
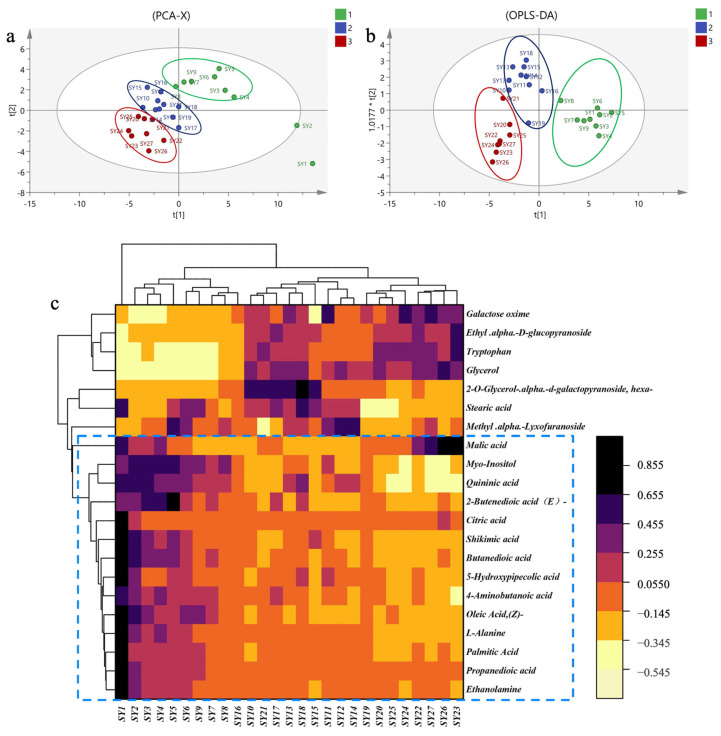
The primary metabolites in MLs at different growth stages based on GC-MS. (**a**): PCA score plots; (**b**): OPLS-DA score plots. In these plots, samples from the first growth stage are highlighted in green, those from the second growth stage are shown in blue, and those from the third growth stage are marked in red. (**c**) Cluster analysis.

PCA was conducted using 46 compounds as independent variables. As depicted in Figure 2a, the cumulative variance interpretation parameter R2X and prediction ability parameter Q2 were found to be 0.921 and 0.391, respectively. These values indicate that the PCA model exhibited strong discriminatory and predictive capabilities. The samples could be broadly categorized into three groups, corresponding to distinct growth stages. The PCA results underscored the significant variations in the identified compound content across different growth stages of MLs.

The OPLS-DA (Figure 2b) model demonstrated cumulative interpretation parameters R2Y and Q2 of 0.812 and 0.623, respectively. These results suggest that the established model possesses robust stability and predictive ability.

To visualize the differences in metabolic profiles of MLs at different growth stages, we conducted a screening of differentially abundant metabolites based on variable importance in projection values (VIP > 1) and *p*-values (*p* < 0.05). This led to the identification of 20 differential chemical markers. Cluster analysis (CA) was performed using OriginPro 2021 (9.8, Origin Lab Corporation, Northampton, MA, USA), employing the inter-group mean connection method and Euclidean distance. The results revealed the division of 27 sample batches into three distinct categories, highlighting significant differences in the relative metabolite content (Figure 2c).

Among these 20 compounds, galactose oxime, ethyl-α-d-glucopyranoside, and glyceric acid tryptophan exhibited significantly lower relative content during the first growth stage compared to the other two growth stages. Conversely, compounds in the blue zone, including myo-inositol, quininic acid, 2-butenedioic acid (*E*)-, citric acid, shikimic acid, butanedioic acid, 5-hydroxypipecolic acid, 4-aminobutanoic acid, oleic acid (*Z*)-, ethanolamine, propanedioic acid, and L-alanine, showed significantly higher relative content during the first growth stage compared to the last two growth stages.

### 2.2. Secondary Metabolic Analysis

#### 2.2.1. UPLC–Quadrupole Time-of-Flight (QTOF)–MS Data and Molecular Networking (MN) Analysis

**Figure 3:**
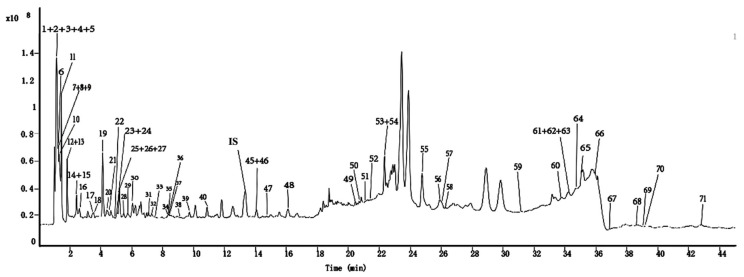
Total ion flow diagram of MLs obtained by HPLC-QTOF-MS in positive ion mode.

**Figure 4:**
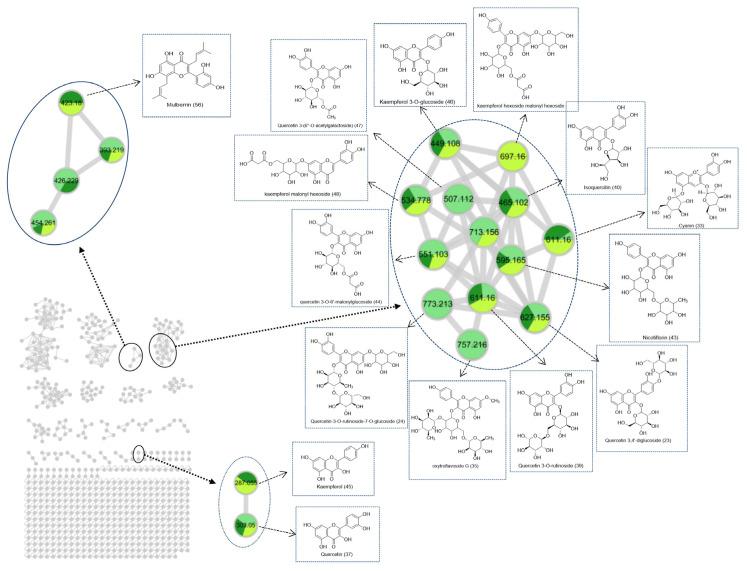
Molecular networking diagrams of MLs at different growth stages.

In this investigation, a secondary metabolite analysis was performed on 27 batches of MLs at various growth stages utilizing UPLC-QTOF-MS. Figure 3 illustrates the total ion chromatogram for the MLs, while Table 2 provides the list of tentatively identified compounds. A network based on spectral similarity provides a visual tool for examining tandem mass spectrometry data. This facilitates the annotation of compounds while also allowing the observation of features distributed across various samples [19]. The creation of the MN was based on the similarity of MS/MS spectra, as visually presented in Figure 4. In the figure, the node size is indicative of the relative quantity of the respective compound, and each node is depicted as a pie chart [20]. This diagram emphasizes the primary cluster of flavonoids, which encompasses compounds such as quercetin (peak 37, *m*/*z* 303.05, [M + H]^+^), isoquercetin (peak 40, *m*/*z* 465.1206, [M + H]^+^), kaempferol (peak 45, *m*/*z* 287.0548, [M + H]^+^), and others [21,22]. Within each type of flavonoid structure, common substituents on the A and B rings consist of hydroxyl, methyl, and methoxy groups. The loss of these neutral fragments represents the fundamental fragmentation pathway for flavonoids. Most of the flavonoid glycosides were *O*-glycosides, as indicated by the neutral losses of rhamnopyranosyl (*m*/*z* 146) and glucose residues (*m*/*z* 162). MS/MS fragments of [M-162]^+^ were generated due to typical C-glycoside cleavages, suggesting that glycosyl groups are linked to the flavonoid glycosides through C-glycosidic bonds. For instance, quercetin 3,4-diglycosides (peak 23, *m*/*z* 627.1554, [M + H]^+^) represent glycosides linked to quercetin aglycones through C3 and C4 bonds [23].

#### 2.2.2. Secondary Metabolites of MLs at Different Growth Periods

Isochlorogenic acid A served as the reference peak during the method validation for metabolites in ML extracts. The relative peak area (RPA) for each compound was calculated using the area normalization method. Subsequently, we employed the multivariate statistical analysis software SIMCA 14.1 to conduct PCA and OPLS-DA to identify differential metabolites.

**Figure 5:**
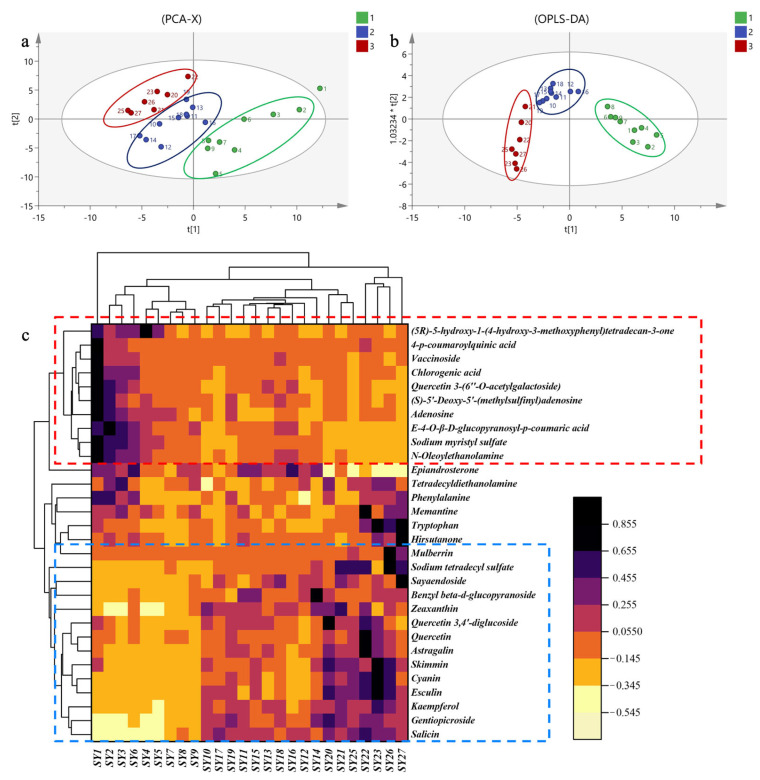
The secondary metabolites in MLs at different growth stages analyzed by HPLC-QTOF-MS. (**a**): PCA score plots; (**b**): OPLS-DA score plots. In these plots, samples from the first growth stage are highlighted in green, those from the second growth stage are shown in blue, and those from the third growth stage are marked in red). (**c**): Cluster analysis.

PCA was conducted on MLs at different growth stages, yielding cumulative variance explanatory (R2X) and predictive (Q2) ability parameters of 0.688 and 0.494, respectively. These values indicate the effectiveness of the PCA model in discriminating between samples. As depicted in Figure 5a, the 27 batches of samples from various growth stages were categorized into three distinct regions, suggesting notable differences in metabolite content among MLs at different growth stages.

Furthermore, OPLS-DA was employed to establish a partial least squares discriminant analysis model. The cumulative explanatory power parameters R2Y and Q2 were 0.817 and 0.682, respectively, signifying the stability and predictive capability of the model (Figure 5b). The OPLS-DA score plot also revealed a similar categorization of MLs at different growth stages into three distinct groups, consistent with the PCA results.

Differential metabolite screening in MLs at different growth stages was carried out by considering variables with a VIP score greater than 1 and a *p*-value less than 0.05. Consequently, 30 differential chemical markers were identified. Cluster analysis was performed using Origin software 2021 (Northampton, MA, USA), employing the inter-group mean connection method and Euclidean distance.

The clustering heatmap (depicted in Figure 5c) demonstrated that MLs at different growth stages could be clearly distinguished based on the clustering patterns of the identified compounds. These findings align with the results obtained from PCA and OPLS-DA analyses. Among the 30 compounds, those in the red zone (4-*p*-coumaroylquinic acid, vaccinoside, chlorogenic acid, quercetin 3-(6″-*O*-acetylgalactoside), (*S*)-5′-deoxy-5′-(methylsulfinyl) adenosine, adenosine, (*E*)-4-*O*-β-d-glucopyranosyl-*p*-coumaric acid, sodium myristyl sulfate) exhibited significantly higher levels during the first growth stage compared to the last two growth stages. Conversely, compounds in the blue zone (tryptophan, hirsutanone, sodium tetradecyl sulfate, salicin, gentiopicroside, kaempferol, esculin) displayed significantly lower levels during the first growth stage in comparison to the last two growth stages.

### 2.3. The Determination of Six Compounds from MLs by HPLC Analysis

The validation of the HPLC method encompassed the determination of parameters including linearity, repeatability, precision, stability, and recovery.

#### 2.3.1. Linearity and Method Validation

A set of standard solutions containing six compounds was freshly formulated in methanol to establish the linear range of the analytes. The outcomes of calibration were compiled in Table 3, revealing strong correlations between the peak area (y) and the concentration of the tested compounds (x) (*r* > 0.9995) within the specified test ranges. This observation affirmed the acceptability and exceptional sensitivity of the analytical method.

#### 2.3.2. Simultaneous Quantitative Analysis of Six Constituents of MLs

**Figure 6:**
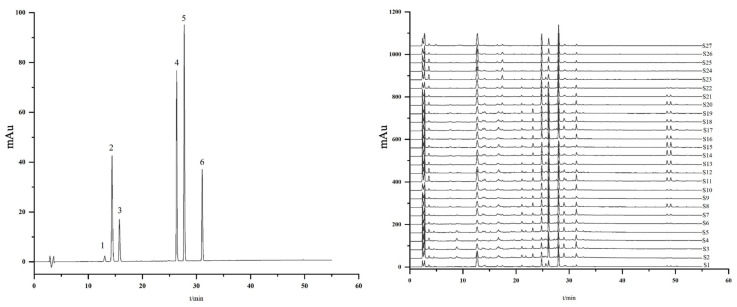
HPLC chromatogram of 27 batches of MLs. 1. Mulberroside A; 2. Chlorogenic acid; 3. Cryptochlorogenic acid; 4. Rutin; 5. Isoquercitrin; 6. Oxidized resveratrol.

**Figure 7:**
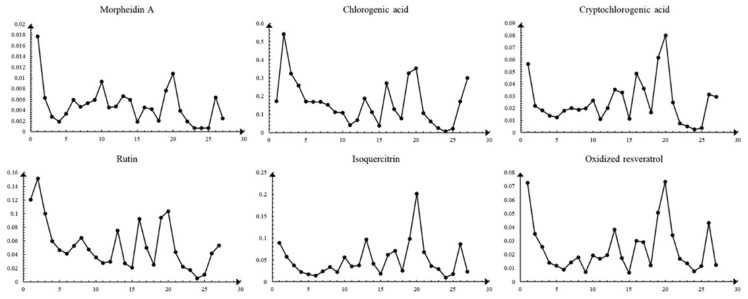
Changes in six compounds in MLs at different growth stages.

HPLC was employed to assess the variations in six components of MLs across different growth stages, encompassing mulberroside A, chlorogenic acid, cryptochlorogenic acid, rutin, isoquercitrin, and oxidized resveratrol. The observed changes in the content of these six compounds in MLs align with the findings from GC-MS and UPLC-QTOF-MS. Specifically, the compositional changes can be roughly categorized into three stages, with the highest content of these compounds observed at the Y20 (Table 4) stage. All results are shown in Figure 6 and Figure 7.

### 2.4. Antioxidant Activities of MLs at Different Growth Stages

**Figure 8:**
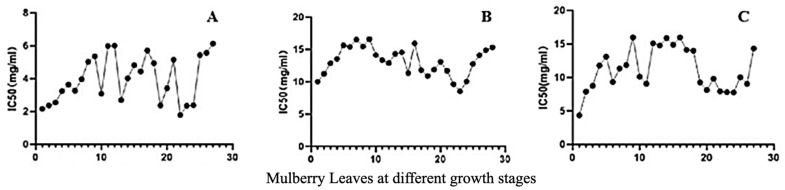
Antioxidant activity changes of MLs at different growth stages: (**A**) IC_50_ of DPPH free radicals; (**B**) IC_50_ of hydroxyl radicals; (**C**) IC_50_ of ABTS free radicals.

Based on the data presented in Figure 8A, it can be observed that the IC_50_ (half maximal inhibitory concentration values), which represent the DPPH radical scavenging activity, exhibited notable variations during different stages of ML growth. Specifically, at the early growth stage of MLs, a lower IC_50_ value indicated a strong antioxidant activity. However, as the IC_50_ value increased, the antioxidant activity of MLs displayed irregular fluctuations, and around the Y23 stage, the antioxidant activity increased and then decreased.

Similarly, Figure 8B also reveals fluctuations in the scavenging capacity of MLs for hydroxyl free radicals over the course of their growth. Initially, during the early growth stages, MLs exhibited a strong ability to scavenge hydroxyl free radicals, which subsequently decreased and displayed irregular fluctuations. The scavenging ability reached its peak at Y23, followed by a decrease. Notably, these observations regarding the antioxidant activity were consistent with the trends observed for DPPH free radical scavenging.

Furthermore, Figure 8C demonstrates that MLs exhibited a low IC_50_ value and a high clearance rate during the early growth stage, indicating strong clearance of ABTS free radicals. However, as the growth stage progressed, the clearance rate of MLs for ABTS free radicals exhibited irregular fluctuations. Similarly, IC_50_ values decreased, and the clearance rate was high at the Y23 stage.

### 2.5. The Inhibitory Effects of MLs at Different Growth Stages on α-Glucosidase Activities

α-Glucosidase is a group of enzymes located in the brush border membrane of the small intestine. It catalyzes the hydrolysis of various polysaccharides, including starch, sucrose, and maltose, as well as oligosaccharides and disaccharides present in food, into absorbable monosaccharides, such as glucose and fructose. α-Glucosidase inhibitors competitively hinder the activity of α-glucosidase. They delay the conversion of polysaccharides, oligosaccharides, and disaccharides into monosaccharides, thereby regulating postprandial blood glucose levels and preventing sharp increases in blood glucose levels after meals [67].

**Figure 9:**
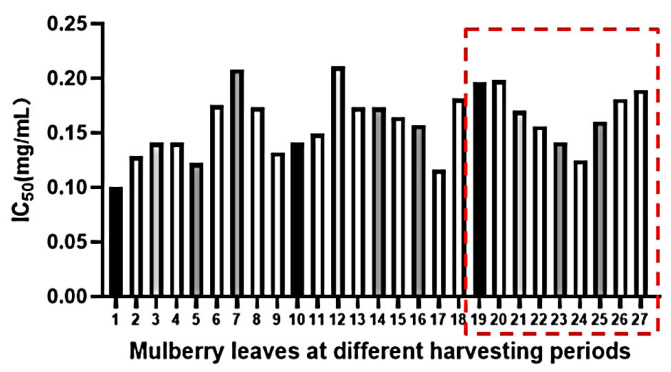
IC_50_ of α-Glucosidase in MLs at different growth periods.

The experimental method for assessing α-glucosidase activity is based on the reaction between α-glucosidase and PNPG, resulting in the production of *p*-nitrophenol. When a sample solution is introduced into the system, it inhibits the activity of α-glucosidase within the sample, subsequently reducing the production of *p*-nitrophenol and leading to a decrease in absorbance. As depicted in Figure 9, during the early stage of leaf growth, MLs exhibited a lower IC_50_ value, indicating a stronger inhibitory effect on α-glucosidase. The red dashed line illustrates a decrease in the IC_50_ value before and after the traditional Chinese solar term ‘Frost’s Descent’, followed by an increase in the later period. This suggests that MLs harvested both before and after the period of frost’s descent possess enhanced inhibition of α-glucosidase activity. 

## 3. Materials and Methods

### 3.1. Plant Materials and Chemicals

#### 3.1.1. Plant Materials

A total of 27 batches of MLs, representing various growth periods, were gathered from the medicinal plant garden at Hebei University of Chinese Medicine (coordinates: 114°20′47.16″ E, 38°3′24.27″ N, elevation 118.23 m above sea level). To ensure uniformity in sampling, leaves were collected simultaneously from multiple trees within the same area and at different heights. Three replications of the samples were collected and analyzed. These samples were subsequently authenticated by Prof. Dan Zhang. The collection dates for the 27 samples are detailed in Table 4. All specimens were securely stored at Hebei University of Chinese Medicine.

#### 3.1.2. Chemicals

Derivatization reagents including anhydrous pyridine (high-purity grade) and the internal standard arabitol, sourced from Shanghai Aladdin Biochemical Technology Co., Ltd. (Shanghai, China), as well as methoxyamine hydrochloride (GC-grade) and *N*-methyl-*N*-trimethylsilyl-trifluoroacetamide (MSTFA, GC-grade) obtained from Sigma-Aldrich (St. Louis, MO, USA), were utilized in the study. Isochlorogenic acid B (MUST-20031602) was acquired from Chengdu Must Bio-technology Co., Ltd. (Chengdu, China). Standard substance, such as mulberroside A (PRF9070342), chlorogenic acid (110753-201415), cryptochlorogenic acid (PRF9091941), rutin (100080-202012), isoquercitrin (PRF9092807), oxidized resveratrol (MUST-20031602), and xylitol (100463-202003) were obtained from Chengdu Biopurify Phytochemicals Ltd. (Chengdu, China), and 2,4,6-Tripyridyl-s-triazine, K_2_S_2_O_8_, 2,2′-azinobis-(3-ethylbenzthiazoline-6-sul phonae) (ABTS), and 2,2-diphenyl-1-picryl-hydrazyl (DPPH) were obtained from Aladdin Biochemical Technology Co. Ltd. (Shanghai, China). HPLC grade methanol and acetonitrile were purchased from Fisher Scientific Co., Ltd. (Pittsburgh, PA, USA). LC-MS-grade methanol, acetonitrile, and formic acid were obtained from Fisher Scientific (Pittsburgh, PA, USA), while ultrapure water was produced using a Synergy water purification system (Millipore, Billerica, MA, USA). All other chemicals and reagents used were of analytical grade.

### 3.2. Sample Preparation for MS and HPLC Analyses

All samples were subjected to freeze-drying and subsequent grinding to obtain a fine powder. The resulting powder was sieved through a 40-mesh screen. An exact 0.5 g of ML powder was carefully placed into a conical bottle with a stopper, followed by the precise addition of 17.5 mL of 80% ethanol. Ultrasonication was performed for a duration of 45 min while maintaining the water temperature at a constant 60 °C. The mixture was then cooled to room temperature and weighed to compensate for any weight loss. Subsequently, centrifugation was conducted at 13,000 r·min^−1^ for 10 min, and the resulting supernatant was collected.

To ensure the stability of the analytical method, quality control (QC) samples were prepared by equally combining powders from all samples. 

#### 3.2.1. GC-MS Analysis

For sample preparation, 100 μL of the extract and 20 μL of arabitol solution (0.8 mg/mL) were combined in screw-cap vials and subjected to evaporation under a nitrogen gas stream at 40 °C until complete dryness. The derivatization of primary metabolites of the extracted samples was assessed using a method previously described with slight modifications [15,68,69]. An amount of 20 μL of methoxyamine in pyridine (20 mg/mL) was added to the sample tube, followed by incubation at 37 °C for 150 min in a dry bath. Subsequently, for silylation, 80 μL of *N*-methyl-*N*-(trimethylsilyl)-trifluoroacetamide (MSTFA) was added to the mixture and incubated at 37 °C for 150 min under the same conditions as previously mentioned. Finally, the sample underwent centrifugation at 13,000 r·min^−1^ for 10 min, and the resulting supernatant was collected for GC-MS analysis.

GC-MS analysis was performed using an Agilent 7890B GC system coupled with a 5977B MSD mass detector (Agilent Technologies, Santa Clara, CA, USA). The GC-MS instrument was equipped with an Agilent HP-5MS 5% phenyl methyl siloxane capillary column (30 m × 0.25 mm, 0.25 µm, Agilent, Santa Clara, CA, USA). A 1 µL aliquot of the prepared supernatant solution was introduced in split mode with a split ratio of 10:1, at an injection temperature of 250 °C. The oven temperature program initiated at 60 °C, then ramped up to 130 °C at a rate of 20 °C·min^−1^, held for 1 min, further increased to 150 °C at a rate of 5 °C·min^−1^, and then elevated to a maximum of 220 °C at a rate of 3 °C·min^−1^, with a 1-min hold. Following this, the temperature was raised to 255 °C at a rate of 5 °C·min^−1^ and maintained for 5 min. 

Mass spectrometry parameters involved the utilization of an electron ionization ion source (EI) with an ion energy of 70 eV. The interface temperature, ion source temperature, and quadrupole temperature were maintained at 250 °C, 230 °C, and 150 °C, respectively. The scanning mass range for the total ion chromatogram (TIC) extended from *m*/*z* 50 to 500, and a solvent delay time of 3 min was applied.

#### 3.2.2. UPLC-MS Analysis

Analysis with UPLC-Q-TOF/MS was carried out using an Agilent 1290 Infinity II system connected to an Agilent 6545 quadrupole time-of-flight mass spectrometer system (Q-TOF-MS) (Agilent Technologies, Santa Clara, CA, USA), which featured an electrospray ionization interface.

Chromatographic separation was carried out using an Agilent ZORBAX SB-18 column (4.6 × 50 mm, 1.8 μm, Santa Clara, CA, USA). The binary gradient elution system consisted of acetonitrile (B) and water with 0.1% formic acid (A). Separation was performed at a flow rate of 0.4 mL·min^−1^, following this gradient program: 0–5 min, from 9% to 15% B; 5–16 min, from 15% to 24% B; 16–20 min, from 24% to 52% B; 20–30 min, from 52% to 55% B; 30–33 min, from 55% to 70% B. The sample injection volume was 1 µL, and the column temperature was maintained at 25 °C.

For mass spectrometry acquisition, the parameters were configured as follows: drying gas (N_2_) temperature set at 320 °C; sheath gas (N_2_) temperature at 350 °C; drying gas (N_2_) flow maintained at 10.0 L·min^−1^; sheath gas (N_2_) flow at 11 L·min^−1^; nebulizer gas (N_2_) pressure maintained at 35 psi; capillary voltage held at 3500 V; fragmentor voltage at 135 V; and collision energy set to 40 eV. The analysis was performed in positive mode, covering a mass range from *m*/*z* 100 to 1000 Da. Subsequently, the data obtained were processed using MassHunter Qualitative Analysis Software Version B.10.00 (Agilent Technologies, Santa Clara, CA, USA).

#### 3.2.3. HPLC Analysis

HPLC analysis was carried out using a Shimadzu LC-2030 3D system equipped with a photodiode-array detector (Shimadzu, Kyoto, Japan). Chromatographic separation was achieved on an Agilent ZORBAX Eclipse XDB-C18 column (5 μm, 4.6 × 250 mm, Agilent Technologies, Santa Clara, CA, USA) while maintaining the column temperature at 30 °C. Detection was performed at a wavelength of 254 nm, and a 10 μL injection volume was used. The mobile phase consisted of a gradient mixture of water containing 0.1% formic acid (A) and acetonitrile (B), flowing at a rate of 1 mL·min^−1^. The gradient elution program was as follows: 0–5 min: 9% B, 5–13 min: 9–13% B, 13–25 min: 13–22% B, 25–55 min: 22–48% B, 55–65 min: 48–65% B, and 65–75 min: 65–79% B.

### 3.3. GC-MS Profiling and Modeling of Silylated Primary Metabolites

We validated silylation following the protocol described in our previous work [69]. Metabolite identification in MLs involved comparing their retention indices (RI) to *n*-alkanes (C7–C40) and aligning their masses with entries in NIST17 library. After normalization, the dataset was imported into SIMCA software (version 14.0, Umetrics, Umea, Sweden) for orthogonal principal component analysis (PCA) and partial least squares discriminant analysis (OPLS-DA).

### 3.4. Analysis, Modeling, and Quantification of the UPLC-QTOF-MS Dataset

#### 3.4.1. GNPS Molecular MS/MS Network

MN construction utilized UPLC-QTOF MS/MS data. All MS/MS data files were converted to 32-bit mzXML format with ProteoWizard software (https://proteowizard.sourceforge.io, accessed on 1 December 2023). These transformed files were then transferred to the GNPS platform (https://gnps.ucsd.edu, accessed on 1 December 2023) via WinSCP (https://winscp.net, accessed on 1 December 2023) to initiate MN generation following an online workflow [19]. The MN parameters were set as follows: a minimum cosine score of 0.70, a requirement of at least 6 matched peaks, a tolerance of 0.02 Da for both parent mass and fragments, a maximum connected component size of 100, a minimum cluster size of 1, and the exclusion of the run MScluster tool. Access to the resultant MN and its related parameters can be found through this link: (http://gnps.ucsd.edu/ProteoSAFe/status.jsp?task=3902109bbf36453ca44f4fa5a553c85b, accessed on 1 December 2023). Metabolites were identified based on their molecular formula and fragmentation pattern, with reference to previously reported data, public literature, libraries, and databases. The obtained results were then exported for visualization using Cytoscape 3.8.2 software (La Jolla, CA, USA).

#### 3.4.2. Data Processing and Statistical Analysis

The LC-MS data acquisition was conducted using the MassHunter Workstation (Agilent Technologies). After data normalization, the dataset was imported into SIMCA software (version 14.0, Umetrics, Umea, Sweden) for PCA and OPLS-DA. Additionally, the data for different growth periods were designated as a Y variable and subjected to statistical analysis, which included partial least squares (PLS), partial least squares discriminant analysis (PLS-DA), and OPLS-DA. Variable importance in projection (VIP) values obtained from the OPLS-DA analysis were employed for marker compound identification in MLs during storage. Cluster analysis (CA) was carried out using OriginPro 2021 (9.8, Origin Lab Corporation, Northampton, MA, USA).

### 3.5. Standard Substance Solution and Sample Solution Preparation

A known quantity of standard substances was utilized to create a composite solution in methanol, which included mulberroside A, chlorogenic acid, cryptochlorogenic acid, rutin, isoquercitrin, and oxidized resveratrol, at concentrations of 0.006, 0.0944, 0.032, 0.086, 0.064, and 0.052 mg/mL, respectively.

For the sample preparation, 0.5 g of ML powder was precisely weighed and transferred to a conical vial. Then, 17.5 mL of 80% ethanol was accurately added. The mixture underwent ultrasonication for 45 min, maintaining a constant water temperature of 60 °C. After cooling to room temperature, the mass was re-measured to account for any weight change. Subsequently, centrifugation was performed at 13,000 rpm for 10 min, and the resulting supernatant was collected as the test sample solution.

### 3.6. Determination of Antioxidant Activities at Different Growth Stages of MLs

#### 3.6.1. Inhibition of 1,1 diphenyl 2-picryl hydrazine (DPPH)

The DPPH scavenging activity of the extracted samples was assessed using a method previously described with slight modifications [70]. Extracts from 27 batches of ML samples were diluted to concentrations of 2 mg·mL^−1^, 3 mg·mL^−1^, 4 mg·mL^−1^, 5 mg·mL^−1^, 6 mg·mL^−1^, and 7 mg·mL^−1^, respectively. Different concentrations of ML extract solutions were accurately mixed with 180 μL of DPPH-ethanol solution, followed by incubation for 30 min at 37 °C in the absence of light. Absorbance was then measured at 517 nm. The scavenging rate was calculated using the following formula:

DPPH scavenging rate (%) = AA% = [1 − (A − A0)/B] × 100%

where A corresponds to the absorbance value of the solution with the added sample, A0 represents the control group lacking the DPPH solution, and B denotes the blank group without the sample.

#### 3.6.2. Inhibition of Hydroxyl Radical

To assess the hydroxyl radical scavenging capacity, 27 batches of ML samples were diluted to various concentration gradients. A mixture of 30 μL hydrogen peroxide solution, 30 μL salicylic acid solution, and 30 μL ferrous sulfate solution was added to 120 μL of the ML sample extraction solution. The mixture was incubated for 30 min in the absence of light, and the absorbance was subsequently measured at 517 nm.

The hydroxyl radical scavenging rate was calculated using the following formula:

Hydroxyl radical scavenging rate (%) = AA% = [1 − (A − A0)/B] × 100%

where A represents the absorbance value of the sample solution, A0 denotes the control group without hydrogen peroxide solution, and B signifies the blank group without sample addition.

#### 3.6.3. ABTS Radical Scavenging Activity

The ABTS cation (ABTS^+•^) radical inhibition assay was performed with minor adjustments, following a previously documented method [70]. Initially, ABTS was dissolved in deionized water to attain a concentration of 7 mM. ABTS^+•^ was generated by reacting the ABTS solution with potassium persulfate, reaching a final concentration of 2.45 mM, and allowing this mixture to stand at room temperature in darkness for 12–16 h before application. In this investigation, the ABTS^+•^ solution was thoroughly mixed with distilled water until the absorbance at 734 nm stabilized at 0.70 ± 0.002. Subsequently, 20 μL of varying extract concentrations were combined with 180 μL of the prepared ABTS^+•^ solution and incubated for 10 min. The resulting reaction solutions were then assessed using a PerkinElmer VICTOR Nivo Multimode Plate Reader (PerkinElmer Inc., Mountain View, CA, USA) with excitation and emission wavelengths set at 734 nm. The ABTS^+•^ scavenging rate was calculated using the following formula:

ABTS^+•^ scavenging rate (%) = AA% = [1 − (A − A0)/B] × 100%

where A represents the absorbance value of the solution with the added sample, A0 is the control group without ABTS^+•^ solution, and B is the blank group without the sample.

### 3.7. Determination of α-Glucosidase Inhibitory Activity at Different Growth Stages of MLs

The α-glucosidase inhibitory activity was determined following the protocol outlined by Guo et al. [71]. In a 96-well plate, 75 µL of PBS was added to all wells. Subsequently, 20 µL of ML sample solution at various concentrations was added to the control and background groups, while 20 µL of PBS was added to the control and background groups. Following this, 65 µL of α-glucosidase was added to the sample and control groups, and 65 µL of PBS was added to the blank and background groups. The plate was then incubated at 37 °C for 10 min. After incubation, 30 µL of *p*-nitrophenyl α-d-glucopyranoside (PNPG, Sigma-Aldrich Ltd., Shanghai, China) was added to all groups, followed by another 20 min of incubation at 37 °C. Finally, 50 µL of Na_2_CO_3_ was added to each well to halt the reaction. The absorbance was measured at 405 nm using the PerkinElmer VICTOR Nivo Multimode Plate Reader (PerkinElmer Inc., Mountain View, CA, USA), and the α-glucosidase inhibition rate was calculated using the following formula:

Inhibition (%) = [1 − (A_a_ − A_b_)/A_c_ − A_d_] × 100%

where A_a_ is the sample group, A_b_ is the blank sample group without α-glucosidase, A_c_ is the control group without sample, and A_d_ is the blank group without sample and α-glucosidase.

IC_50_ values were calculated using GraphPad Prism 9 (GraphPad Software, La Jolla, CA, USA).

## 4. Conclusions

The current study employed a non-targeted metabolomics approach based on GC-MS to determine changes in primary metabolites and UPLC-QTOF-MS to identify changes in secondary metabolites in MLs at different growth stages. Metabolite differences indicated that MLs at various growth stages could be roughly categorized into three stages. Samples 1–9 represented the initial growth stage, samples 10–19 corresponded to the intermediate growth stage, and samples 20–27 denoted the final growth stage. Importantly, significant changes in metabolite content occurred during these three growth stages, aligning with China’s 24 solar terms. The first growth stage occurred before the summer solstice, the second growth stage spanned from the summer solstice to the autumnal equinox, and the third growth stage occurred after the autumnal equinox. Seasonal divisions serve as crucial nodes symbolizing climate change in China. Variations in temperature and climate across different seasons greatly influenced metabolite changes. In vitro activity studies revealed significant variations in antioxidant activity and α-glucosidase inhibition among 27 batches of MLs at different growth periods. Enhanced activity was observed in ML samples at the beginning of the growth period and around the time of frost, aligning with the traditional harvest timing of ML medicinal herbs during the period of ‘Frost’s Descent’ in China. This study provides experimental evidence for the rational harvest of ML medicinal herbs and, concurrently, offers technical support for the development and utilization of MLs.

## Figures and Tables

**Table 1:** Identification of silylated primary metabolites from MLs by GC-MS.

No.	*t_R_*/min	Metabolite Name	*m*/*z*	Molecular Formula
1	3.976	Liactic Acid, 2TMS derivative	219.1	C_9_H_22_O_3_Si_2_
2	4.096	Glycolic acid, 2TMS derivative	218.1	C_8_H_20_O_3_Si_2_
3	4.215	L-Valine, TMS derivative	217	C_8_H_19_NO_2_Si
4	4.355	L-Alanine, 2TMS derivative	232.1	C_9_H_23_NO_2_Si_2_
5	4.744	Hydracrylic acid, 2TMS derivative	235.1	C_9_H_22_O_3_Si_2_
6	5.445	Propanedioic acid, 2TMS derivative	248.1	C_9_H_20_O_4_Si_2_
7	5.626	Valine, 2TMS derivative	246.1	C_11_H_27_NO_2_Si_2_
8	5.823	Urea, 2TMS derivative	233.1	C_7_H_20_N_2_OSi_2_
9	6.316	Ethanolamine, 3TMS derivative	262.1	C_11_H_31_NOSi_3_
10	6.415	Glycerol, 3TMS derivative	314.11	C_12_H_32_O_3_Si_3_
11	6.669	Niacin, TMS derivative	301	C_9_H_13_NO_2_Si
12	6.778	L-Proline, 2TMS derivative	314.1	C_11_H_25_NO_2_Si_2_
13	6.944	Butanedioic acid, 2TMS derivative	299	C_10_H_22_O_4_Si_2_
14	7.313	Glyceric acid, 3TMS derivative	322.1	C_12_H_30_O_4_Si_3_
15	7.452	2-Butenedioic acid (*E*)-, 2TMS derivative	299	C_10_H_20_O_4_Si_2_
16	7.805	Serine, 3TMS derivative	306.1	C_12_H_31_NO_3_Si_3_
17	8.288	L-Threonine, 3TMS derivative	320.1	C_13_H_33_NO_3_Si_3_
18	8.407	Ethanolamine, 3TMS derivative	299	C_11_H_31_NOSi_3_
19	8.952	Beta-Alanine, 3TMS derivative	299.1	C_12_H_31_NO_2_Si_3_
20	10.265	Malic acid, 3TMS derivative	350.1	C_13_H_30_O_5_Si_3_
21	10.924	Pipecolic acid, 2TMS derivative	258.1	C_12_H_27_NO_2_Si_2_
22	11.095	4-Aminobutanoic acid, 3TMS de rivative	332.1	C_13_H_33_NO_2_Si_3_
23	12.849	5-Hydroxypipecolic acid, 3TMS derivative	360.2	C_15_H_35_NO_3_Si_3_
24	13.352	L-Glutamic acid, 3TMS derivative	363.11	C_14_H_33_NO_4_Si_3_
25	13.430	L-Phenylalanine, 2TMS derivative	331.1	C_15_H_27_NO_2_Si_2_
26	14.748	Asparagine, 3TMS derivative	348.2	C_13_H_32_N_2_O_3_Si_3_
27	15.791	D-Lyxose, 4TMS derivative	333.2	C_17_H_42_O_5_Si_4_
28	17.088	Glycerol, 3TMS derivative	394.2	C_12_H_32_O_3_Si_3_
29	18.899	Shikimic acid, 4TMS derivative	462.2	C_19_H_42_O_5_Si_4_
30	19.200	Citric acid, 4TMS derivative	465.2	C_18_H_40_O_7_Si_4_
31	20.606	Quininic acid, 5TMS derivative	462.2	C_22_H_52_O_6_Si_5_
32	21.087	Alloxanic acid, 4TMS derivative	446.2	C_16_H_36_N_2_O_5_Si_4_
33	22.303	Methyl-α-Lyxofuranoside, 3TMS derivative	446.3	C_15_H_36_O_5_Si_3_
34	25.032	Palmitic Acid, TMS derivative	456.1	C_19_H_40_O_2_Si
35	27.237	Myo-Inositol, 6TMS derivative	432.2	C_24_H_60_O_6_Si_6_
36	28.545	Galactose oxime, 6TMS derivative	319.2	C_24_H_61_NO_6_Si_6_
37	29.676	Tryptophan, 4TMS derivative	337.2	C_23_H_44_N_2_O_2_Si_4_
38	29.873	Oleic Acid (*Z*)-, TMS derivative	354.3	C_21_H_42_O_2_Si
39	30.340	L-Rhamnose, 4TMS derivative	361.2	C_18_H_44_O_5_Si_4_
40	30.605	Stearic acid, TMS derivative	356.3	C_21_H_44_O_2_Si
41	34.081	2-*O*-Glycerol-α-d-galactopyranoside, hexa-, TMS	361.2	C_27_H_66_O_8_Si_6_
42	38.689	Ethyl-α-d-glucopyranoside, 4TMS derivative	439.2	C_20_H_48_O_6_Si_4_
43	39.275	1-Monopalmitin, 2TMS derivative	459.3	C_25_H_54_O_4_Si_2_
44	41.268	Sucrose, 8TMS derivative	481.3	C_36_H_86_O_11_Si_8_
45	41.729	Lactose, 8TMS derivative	450.2	C_36_H_86_O_11_Si_8_
46	43.275	Glycerol monostearate, 2TMS derivative	487.3	C_27_H_58_O_4_Si_2_

**Table 2:** Information on 70 compounds in MLs identified by HPLC-Q-TOF-MS.

No.	*t_R_*/min	Precursor Ion Type	Precursor Ion (*m*/*z*)	Formula	Fragment Ions (*m*/*z*)	Identification	Ref.
1	1.06	[M + H]^+^	175.1189	C_6_H_14_N_4_O_2_	130.0973, 116.0701, 70.0651, 60.0556	Arginine	[21,24]
2	1.079	[M + H]^+^	164.0916	C_6_H_13_NO_4_	146.0803, 128.0703, 110.0596, 82.0651, 80.0495, 69.0335	1-Deoxynojirimycin (1-DNJ)	[21,24,23]
3	1.089	[M + H]^+^	156.0420	C_6_H_9_N_3_O_2_	110.0715, 93.0440, 82.0261	Histidine	[21,24]
4	1.172	[M + H]^+^	146.0810	C_6_H_11_NO_3_	128.0701, 100.0755	*N*-isobutyrylglycine	[21]
5	1.179	[M + NH_4_]^+^	360.1504	C_12_H_22_O_11_	101.0232, 71.0491	Sucrose	[25]
6	1.202	[M + H]^+^	116.0707	C_5_H_9_NO_2_	116.0706, 70.0652	Proline	[21,24]
7	1.22	[M + H]^+^	137.9750	C_7_H_7_NO_2_	138.0546, 94.0650, 92.0492, 78.0336	Trigonelline	[26]
8	1.31	[M + H]^+^	314.0920	C_11_H_15_N_5_O_4_S	164.0565, 136.0618, 97.0248	(*S*)-5′-Deoxy-5′-(methylsulfinyl)adenosine	[27]
9	1.308	[M + H]^+^	358.1498	C_16_H_20_O_9_	196.0965, 178.0859, 150.0906	Gentiopicroside	[28]
10	1.375	[M + H]^+^	268.1043	C_10_H_13_N_5_O_4_	137.0646, 136.0617	Adenosine	[29]
11	1.463	[M + H]^+^	132.1018	C_6_H_13_NO_2_	86.0964, 69.0697	Isoleucine	[21,24]
12	1.82	[M + H]^+^	284.0989	C_10_H_13_N_5_O_5_	286.1451, 154.0846, 153.0584, 152.514	Guanosine	[30]
13	1.887	[M + H]^+^	287.1102	C_13_H_18_O_7_	107.0296	Salicin	[31]
14	2.413	[M + H]^+^	166.0862	C_9_H_11_NO_2_	103.0539, 121.0842, 120.0805, 107.0494	Phenylalanine	[24]
15	2.477	[M + H]^+^	120.0809	C_4_H_9_NO_3_	119.0705, 102.0116, 84.9590, 75.9350	l-Threonine	[21]
16	2.536	[M + H]^+^	220.1177	C_9_H_17_NO_5_	184.0949, 116.0323, 90.0548	Pantothenic acid	[21]
17	3.431	[M + K]^+^	367.1500	C_19_H_20_O_5_	131.0680, 103.0388	Hirsutanone	[32]
18	3.452	[M + H]^+^	298.0964	C_11_H_15_N_5_O_3_S	163.0414, 145.0314, 136.0615	Vitamin L2	[21]
19	4.08	[M + H]^+^	205.0973	C_11_H_12_N_2_O_2_	188.07606, 159.0917, 146.0599, 144.0808	Tryptophan	[24]
20	4.25	[M + H]^+^	341.0867	C_15_H_16_O_9_	180.0373, 179.0336, 133.0874	Esculin	[21]
21	4.446	[M + H]^+^	325.0920	C_15_H_16_O_8_	164.0425, 163.0387, 107.0494	Skimmin	[33]
22	4.816	[M − H]^+^	194.1150	C_10_H_12_O_4_	195.1212, 136.0472, 135.0435, 59.0714	3-(4-Hydroxy-3-methoxyphenyl) propionic acid	[34]
23	4.999	[M + H]^+^	627.1554	C_27_H_30_O_17_	466.1064, 465.1033, 304.0536, 303.0498, 85.0281	Quercetin 3,4′-diglucoside	[23]
24	5.08	[M + H]^+^	773.2125	C_33_H_40_O_21_	465.1025, 304.0517, 303.0489, 85.0275	Quercetin-3-*O*-rutinoside-7-*O*-glucoside	[23]
25	5.11	[M + H]^+^	467.1174	C_20_H_20_O_13_	154.0219, 153.0171	Ginnalin A	[35]
26	5.189	[M + H]^+^	355.1027	C_16_H_18_O_9_	163.0388, 145.0281, 135.0438, 117.0334	Chlorogenic acid	[21,36]
27	5.276	[M + NH_4_]^+^	344.1340	C_15_H_18_O_8_	165.0538, 119.0482, 91.0407	*E*-4-*O*-β-d-glucopyranosyl-*p*-coumaric acid	[37]
28	5.575	[M + H-H_2_O]^+^	518.1870	C_25_H_28_O_13_	148.0472, 147.0435, 119.0484, 91.0531	Vaccinoside	[38]
29	5.74	[M + NH_4_]^+^	288.1400	C_13_H_18_O_6_	213.0523, 138.9628	Benzyl beta-d-glucopyranoside	[39]
30	5.983	[M + H]^+^	713.1558	C_30_H_32_O_20_	551.1035, 465.1018, 303.0498	Quercetin 3-*O*-(6″-malonyl-glucoside) 7-*O*-glucoside	[21,23]
31	7.27	[M + H]^+^	611.1604	C_27_H_31_O_16_+	615.1748, 613.1666, 612.1619, 611.1609	Cyanin	[27]
32	7.298	[M + H]^+^	339.1070	C_16_H_18_O_8_	148.0469, 147.0438, 120.0520, 119.0487, 91.0533	4-*p*-Coumaroylquinic acid	[40]
33	7.46	[M + H]^+^	697.1603	C_30_H_32_O_19_	697.1054, 449.1069, 288.0577, 287.0547	Kaempferol hexoside malonyl hexoside	[21,23]
34	8.349	[M + H]^+^	757.2164	C_33_H_40_O_20_	611.1635, 465.0997, 303.0492	Quercetin-3-*O*-rutinoside-7-Orhamnoside	[21,23]
35	8.36	[M + H]^+^	757.2164	C_34_H_42_O_19_	85.0281, 71.0344	Oxytroflavoside G	[41]
36	8.377	[M + NH_4_]^+^	434.2020	C_19_H_28_O_10_	145.0479, 133.0550, 115.0369, 97.0284, 85.0279	Sayaendoside	[42]
37	8.41	[M + H]^+^	303.0500	C_15_H_10_O_7_	257.0420, 229.0490, 165.0149, 153.0172, 137.0226	Quercetin	[21,22]
38	9.457	[M + H]^+^	180.1017	C_12_H_21_N	180.1023, 163.0960, 135.1002, 121.0835	Memantine	[43]
39	9.73	[M + H]^+^	611.1600	C_27_H_30_O_16_	465.9610, 303.0491	Quercetin 3-*O*-rutinoside (rutin)	[21,44]
40	11.741	[M + H]^+^	465.1206	C_21_H_20_O_12_	305.0577, 304.0529, 303.0492	Isoquercitin	[22]
41	13.02	[M + H]^+^	373.2220	C_19_H_32_O_7_	135.1148, 109.1007	Byzantionoside B	[45]
42	13.048	[M + H]^+^	595.1604	C_27_H_30_O_15_	287.0547, 129.0562, 127.0406, 85.0299, 71.0488	Kaempferol-3-*O*-rutinoside	[23]
43	13.084	[M + H]^+^	595.1649	C_27_H_30_O_15_	287.0535, 129.0521, 127.0404, 85.0274, 71.0489	Nicotiflorin	[23]
44	13.151	[M + H]^+^	551.1207	C_24_H_22_O_15_	304.0525, 303.0491, 159.0282, 127.0382, 109.0280	Quercetin 3-*O*-6′-malonylglucoside	[19,46]
45	14.04	[M + H]^+^	287.0548	C_15_H_10_O_6_	213.0570, 165.0185, 153.01732, 121.0267	Kaempferol	[21]
46	14.045	[M + H]^+^	449.1079	C_21_H_20_O_11_	451.3095, 289.0580, 288.0580, 287.0545	Kaempferol 3-*O*-glucoside (astragalin)	[21,23,44]
47	14.809	[M + H]^+^	507.1124	C_23_H_22_O_13_	507.3026, 303.0493	Quercetin 3-(6″-*O*-acetylgalactoside)	[47]
48	16.012	[M + H]^+^	535.1080	C_24_H_22_O_14_	289.0606, 288.0571, 287.0544	Kaempferol malonyl hexoside	[23]
49	20.35	[M + H]^+^	316.3207	C_14_H_29_NaO_4_S	319.1866, 317.3239, 316.3207, 106.0861	Sodium tetradecyl sulfate	[48]
50	20.81	[M + Na]^+^	264.2320	C_14_H_8_O_4_	203.1431	Alizarin	[49]
51	21.11	[M + H]^+^	361.1389	C_21_H_28_O_5_	361.1395, 344.1892, 325.1344, 307.1182	Prednisolone	[23]
52	21.66	[M + H]^+^	291.1953	C_19_H_30_O_2_	273.1829, 161.1278, 147.1172	Epiandrosterone	[50]
53	22.22	[M + K]^+^	225.5670	C_9_H_16_O_4_	125.0944, 123.1161, 97.1005	Azelaic acid	[51]
54	22.277	[M + H]^+^	274.2741	C_16_H_35_NO_2_	274.2740, 256.2633	*N*-Lauryldiethanolamine	[52]
55	24.615	M + ACN + H	432.2378	C_22_H_30_O_6_	135.0799, 119.0852, 107.0861	Prostratin	[53]
56	25.95	[M + H]^+^	423.1803	C_25_H_26_O_6_	423.1795, 311.0543, 241.0496	Mulberrin	[54]
57	26.139	[M + H]+	302.3051	C_18_H_39_NO_2_	302.2048, 284.2936, 106.0851, 102.0911, 88.0755	Tetradecyldiethanolamine	[55]
58	26.216	[M + H]^+^	302.3053	C_18_H_39_NO_2_	302.3050, 284.2935, 106.0861, 88.0752	Ethanol, 2,2′-(tetradecylimino)bis-	[56]
59	31.35	[M + H]^+^	496.3389	C_24_H_50_NO_7_P	478.3274, 258.1112, 184.0726	1-Palmitoyl-sn-glycero-3-phosphocholine	[57]
60	33.871	[M + H]^+^	317.1800	C_14_H_29_NaO_4_S	317.3232, 106.0857	Sodium myristyl sulfate	[58]
61	34.09	[M + H]^+^	322.2739	C_20_H_35_NO_2_	322.2739, 261.2212, 243.2112	α-Linolenoyl ethanolamide	[59]
62	34.23	[M + H]^+^	421.1640	C_25_H_24_O_6_	365.1019, 323.0605	Kuwanon A	[54,60]
63	34.27	[M + H]^+^	326.2687	C_20_H_39_NO_2_	326.3762, 310.0664	*N*-oleoylethanolamine	[61]
64	34.814	[M + K]^+^	470.4199	C_21_H_20_O_10_	283.1673	Isovitexin	[58]
65	34.99	[M + H]^+^	407.1852	C_25_H_26_O_5_	283.0596, 255.0648	Rubraflavone A	[55]
66	36.10	[M + H]^+^	403.2328	C_20_H_34_O_8_	259.1531, 157.0129, 185.0806, 139.0022, 129.0180	Tributyl acetylcitrate	[62]
67	36.95	[M +H]^+^	282.2790	C_18_H_34_O_2_	69.0698, 57.0307, 55.0540	*cis*-Octadecenoic acid	[63]
68	38.90	[M + H]^+^	391.2838	C_24_H_38_O_4_	167.0331, 149.0230, 71.0854, 57.0698	Bis(2-ethylhexyl) phthalate	[64]
69	39.363	[M + H]^+^	284.2942	C_18_H_37_NO	285.2801, 284.2927	*N*,*N*-dimethylpalmitamide	[65]
70	39.47	[M + H]^+^	352.3200	C_21_H_34_O_4_	177.1252, 137.0933	5-hydroxy-1-(4-hydroxy-3-methoxyphenyl) tetradecan-3-one	[66]

**Table 3:** Calibration plots for the six compounds.

Compound	Linearity Range (mg/mL)	Calibration Equation	Correlation Factor (*r*)
Mulberroside A	0.0003~0.006	y = 5,921,740.33 x + 467.35	0.9995
Chlorogenic acid	0.00295~0.0944	y = 9,556,420.73 x + 3459.11	0.9998
Cryptochlorogenic acid	0.001~0.032	y = 10,673,897.94 x + 641.53	0.9999
Rutin	0.00215~0.086	y = 16,281,522.08 x − 332.238	0.9999
Isoquercitrin	0.0016~0.064	y = 27,860,160.12 x − 666.51	0.9999
Oxidized resveratrol	0.0013~0.052	y = 15,236,962.42 x − 989.82	0.9999

**Table 4:** The collected dates information for 27 batches of MLs.

No.	Collecting Time	No.	Collecting Time
Y1	13 April 2018	Y15	23 August 2018
Y2	20 April 2018	Y16	1 September 2018
Y3	27 April 2018	Y17	8 September 2018
Y4	4 May 2018	Y18	15 September 2018
Y5	11 May 2018	Y19	21 September 2018
Y6	25 May 2018	Y20	28 September 2018
Y7	1 June 2018	Y21	5 October 2018
Y8	8 June 2018	Y22	11 October 2018
Y9	22 June 2018	Y23	18 October 2018
Y10	6 July 2018	Y24	29 October 2018
Y11	13 July 2018	Y25	3 November 2018
Y12	23 July 2018	Y26	9 November 2018
Y13	1 August 2018	Y27	15 November 2018
Y14	8 August 2018		

## Data Availability

Data are contained within the article.
